# Irrigation improves weight‐for‐height *z*‐scores of children under five, and Women's and Household Dietary Diversity Scores in Ethiopia and Tanzania

**DOI:** 10.1111/mcn.13395

**Published:** 2022-06-24

**Authors:** Dawit K. Mekonnen, Jowel Choufani, Elizabeth Bryan, Beliyou Haile, Claudia Ringler

**Affiliations:** ^1^ International Food Policy Research Institute (IFPRI) ILRI Compound Addis Ababa Ethiopia; ^2^ IFPRI Washington DC USA

**Keywords:** dietary diversity, Ethiopia, irrigation–nutrition linkages, Tanzania, women and children

## Abstract

Evidence on the potential for agricultural intensification to improve nutrition has grown considerably. While small‐scale irrigation is a key factor driving agricultural intensification in sub‐Saharan Africa, its impact on nutrition has not yet been thoroughly explored. In this study, we assess the impact of adoption of small‐scale irrigation in Ethiopia and Tanzania on household and women's dietary diversity, as well as children's nutrition. We use two rounds of primary data collected from irrigators and nonirrigators in Ethiopia and Tanzania. We used a panel fixed effects econometric approach to control for observed household, women and children specific characteristics as well as observed and unobserved time‐invariant confounding factors. The results show that among Ethiopian households who reported having faced drought, women in irrigating households have higher Women's Dietary Diversity Score (WDDS) compared to women in nonirrigating households. In Tanzania, women in irrigating households have higher WDDS compared to nonirrigators and the impact of irrigation on WDDS more than doubles among households facing drought. In addition, among Tanzanian households who reported having faced a drought shock, irrigating households have higher Household Dietary Diversity Score compared to nonirrigators. Children in irrigating households in Ethiopia have weight‐for‐height *z*‐scores (WHZ) that are 0.87 SDs higher, on average, than WHZ of children in nonirrigating households. In Tanzania, irrigation leads to higher WHZ‐scores in children under‐five among households who reported having experienced a drought in the 5 years preceding the survey. The study shows small‐scale irrigation has a strong effect on households' economic access to food and on nutritional outcomes of women and children.

## Key messages

- Irrigation improves weight‐for‐height *z*‐scores of children under five in Ethiopia by 0.87 SDs.
- Irrigation improves weight‐for‐height *z*‐scores of children under five in Tanzania by 0.62 SDs in households that reported having faced drought.
- Among households that reported having faced drought, irrigation improves women's dietary diversity in Ethiopia (by about 9%) and household dietary diversity in Tanzania (by about 18%).
- Irrigation improves women's dietary diversity in Tanzania by about 7.6% among households who did not report drought, and the impact of irrigation on women's diet more than doubles among households facing drought.

## 1 INTRODUCTION

Evidence on the potential for agricultural interventions to contribute to improved nutrition has grown considerably over the past decade (Ruel et al., 2018). Numerous studies have explored both positive and negative effects of agriculture on nutrition and health (Herforth & Harris, 2014; Hoddinott, 2012; Masset et al., 2012; Ruel & Alderman, 2013). However, while small‐scale irrigation is an important component of agricultural intensification, particularly in sub‐Saharan Africa, the impact on nutrition has not yet been thoroughly explored.

The positive and negative linkages between small‐scale irrigation and food security, nutrition, and health are described in Domènech (2015) and Passarelli et al. (2018) and include five main pathways: (1) a production pathway (through changes in agricultural productivity, crop diversification and the extension of the production season to the dry season), (2) an income pathway (from market sales and employment generation), (3) a water supply pathway (from multiple uses of water, such as for water, sanitation and hygiene (WASH) and livestock rearing), (4) a women's empowerment pathway (through changes in women's control over assets and resources, decision‐making authority and time burden) and (5) irrigation as new vector‐breeding habitat and a source of water pollution from pesticides and agro‐chemicals.

Evidence of these pathways is emerging and currently mixed (Domenèch & Ringler, 2013). We present some findings from the literature for two of the pathways relevant to this paper: the production pathway and the income pathway. Irrigation has been shown to lead to increased production and consumption of nutrient‐rich foods, such as fruits and vegetables in Burkina Faso (Olney et al., 2015), Benin (Alaofè et al., 2016), Uganda (Kabunga et al., 2015), and Ethiopia and Tanzania (Passarelli et al., 2018). However, other research has shown that increases in production diversity do not always increase dietary diversity and that other measures improve dietary diversity in smallholder households more effectively (Sibhatu & Qaim, 2017; Sibhatu et al., 2015). This is especially true in areas with access to markets (Sibhatu & Qaim, 2017), where food is mostly sourced from markets and not own production. In these areas, the income pathway may be more relevant. The literature from various contexts shows that irrigation contributes to increased income from the sale of irrigated crops as well as increased irrigation‐related employment (Burney & Naylor, 2012; Namara et al., 2011; Passarelli et al., 2018). Ex‐ante analyses at the regional scale show considerable potential for further economic gains through the expansion of small‐scale irrigation in sub‐Saharan Africa (Xie et al., 2014; You et al., 2011).

Tanzania and Ethiopia have significant potential for surface and groundwater irrigation, but only a limited number of rural households have access to improved water management and irrigation technologies due to various policy, institutional, technology and market constraints (Amani, 2005; Awulachew et al., 2010). While 14% of Tanzania's 13.5 million hectares of cultivated area could potentially be irrigated, only 2% is currently equipped for irrigation. Similarly, only 5% of Ethiopia's cultivated area of 16 million hectares is currently equipped for irrigation, although 17% of area has the potential to be irrigated (FAO, 2016). Like other countries in the region, Ethiopia's irrigation investments have historically focused on large‐scale schemes with mixed results. Of its irrigated land, 19%, 28% and 53% are small‐scale (<200 hectares [ha]), medium‐scale (200–3000 ha) and large‐scale (>3000 ha) systems (Awulachew et al., 2007).

This article presents results of the impact of irrigation on household and individual‐level nutrition outcomes using panel data collected as part of the Feed the Future Innovation Laboratory for Small Scale Irrigation (ILSSI) in Ethiopia and Tanzania, supported by the United States Agency for International Development (USAID).

## 2 METHODS

### 2.1 Programme description

'The ILSSI project identified locally appropriate irrigation technologies, practices and other complementary tools, and worked with farm households in selected communities in Ethiopia, Ghana and Tanzania to assess costs, benefits and environmental implications. The analysis in this paper focuses on Ethiopia and Tanzania as the research design is similar for these two countries. A range of technologies and practices were promoted, including manual and motor pumps, drip kits, in situ water harvesting, and scheduling tools, such as wetting front detectors that monitor soil moisture. Farmers selected their preferred technologies from a range of options identified through a process of stakeholder consultation that considered factors such as water availability (both ground and surface water), size of landholding and livelihoods. Farmers already engaged in irrigation activities adopted new or different technologies or practices, while other farmers took up irrigation for the first time. The intervention sites were not selected at random but based on scoping work done by ILSSI partners.

### 2.2 Study design and participants

Because the interventions were not implemented in a systematic way, with farmers selecting different irrigation technologies from a menu of options, the study was broadly designed to evaluate the differences between irrigators (any technology or method) and nonirrigators, irrespective of participation in the programme. In both Ethiopia and Tanzania, irrigators (including those participating in the programme) and nonirrigators were selected from the ILSSI intervention communities and other communities within the same districts that were similar based on observable characteristics (e.g., irrigation suitability, population size and market access). We control for the main source of potential selection of households in the ILSSI villages econometrically using household fixed effects. Information on whether the household was irrigating or not was obtained from the local extension office, and later corroborated through a household survey.

In Ethiopia, the sample for the household survey was drawn from the four *woredas* (districts) in which ILSSI interventions took place: Dangila and Bahir‐Dar Zuria *woredas* in Amhara Region, Lemo in SNNPR and Adami Tulu in Oromia Region. Fifteen *kebeles* (villages) were selected from within the chosen *woredas* based on a set of irrigation‐suitability criteria. The irrigation suitability criteria are based on topography (slope), groundwater accessibility, distance to perennial surface water, distance to main river course, proximity to existing irrigation and market access. The details of these variables and the scoring scheme used for the irrigation suitability analysis are provided in Supplementary Information: Appendix B. The number of *kebeles* selected per *woreda* depended on the size of the population living in areas identified as having high irrigation potential within that *woreda*. Within the *woreda*, project intervention *kebeles*, (which were not randomly selected) were included in the sample. The remaining *kebeles* were drawn randomly with probability proportional to size.

In Tanzania, the sample for the household survey is drawn from the two districts in which ILSSI interventions were carried out: Kilosa and Mvomero districts in the Morogoro region. In addition to the two intervention villages (one in each district), 12 additional villages were randomly selected from the two intervention districts based on irrigation suitability that is similar to the intervention villages. Within districts, villages were randomly selected with probability proportional to population size, except for villages in which the ILSSI interventions took place. Adding the two intervention sites brought the total number of villages in the sample to 14.

### 2.3 Sample size

In Ethiopia, 10 households were randomly selected from the list of irrigators and 10 households from the list of nonirrigators for a total of 20 households per *kebele* for a total of 300 households. A further 142 households were added from the ILSSI intervention sites. In Tanzania, 14 households were randomly selected from the list of irrigators, and 14 households from the list of nonirrigators for a total of 28 households per village. In each of the two ILSSI intervention villages, 84 programme participants were also included along with 30 nonirrigators (15 each). This resulted in a total sample of 450 households in Tanzania. The same households were interviewed at baseline and endline. Given the design of the household surveys in both countries, the irrigation data used in the study are not nationally representative, thereby limiting the generalizability of our findings to other regions of the respective country.

### 2.4 Data collection and measures

The baseline and endline household surveys in Ethiopia were completed in November–December 2014 and February 2017, respectively. The difference in the timing of data collection for the two rounds in Ethiopia was due to security and logistical issues related to demonstrations that took place in the study areas between December 2016 and January 2017. In Tanzania, baseline and follow‐up dates were June–July 2015 and June–July 2017, respectively.

All data were collected by trained enumerators using electronic, android‐based tablets using SurveyCTO (a computer‐assisted personal interview software). All surveys were programmed in English and questions directly translated to Amharic and Oromo in Ethiopia and Swahili in Tanzania. Extensive training was provided to enumerators over 2 weeks. Translations of the questions from English were discussed and practiced during the training. A pre‐test was conducted in a non‐ILSSI, noncontrol village for quality assurance purposes directly before the survey. Ethical clearance was obtained from the ethics institutional review board at the International Food Policy Research Institute as well as from Texas A&M (ILSSI lead). Information about the survey was provided by enumerators and informed consent was obtained from adult survey respondents before the interviews were conducted. The head of household (most often male) answered the survey, except for dietary diversity modules which were answered by the primary female. The primary female refers to either the female head of household (when applicable) or the woman who was most knowledgeable about food consumption of the household. Thus, the dietary data used in the study were collected from the woman in the household who had the most knowledge about food preparation and purchases. For child anthropometric measures, informed consent was obtained from parents as well as verbal assent from children. All enumerators collecting anthropometric data were trained using standard WHO guidelines, and measurements were practiced before the survey. All measurements were undertaken by an anthropometrist and an assistant.

### 2.5 Household data collected

Household variables collected include household characteristics (household size, total number of children under 5 years old, age of the primary female, number of years of formal education of primary female, religion), income‐related variables (whether a household received remittances in the previous 12 months, whether the household head was self‐employed, whether a household received income in the form of a gift), and whether the household experienced any shocks in the 5 years before the baseline survey and in the previous 2 years (between the two surveys). The shocks experienced were divided into three categories: (1) drought, (2) storms and floods, and (3) idiosyncratic shocks (death or illness of a family member). For the child‐level models, the explanatory variables of the household model were augmented with child‐specific explanatory variables. The child‐specific variables include child age (6–12, 12–24 and 24–59 months), gender of the child, number of siblings under 5 years old, whether a child was sick in the past 2 weeks, reported size at birth (smaller than usual, usual larger than usual), and whether a child was exclusively breastfed for the first 6 months.

### 2.6 Irrigation and food production

Data on agricultural production were collected for each crop cultivated in the previous 12 months (one dry and one rainy season). Irrigation was defined as a binary variable at the household level as having irrigation on at least one plot during any season. Total land size was calculated to include all cultivated land (in natural logarithmic transformation). Livestock ownership was divided into three binary variables for whether the household owned, raised or produced (1) cows, (2) goats, sheep and pigs, (3) chickens, guinea fowl and ducks.

### 2.7 Dietary diversity

Household‐level dietary diversity was measured using FANTA II Project's Household Dietary Diversity Score (HDDS) (FAO, 2011; Swindale & Bilinsky, 2006). HDDS measures a household's economic access to food, and is the count of the number of food groups consumed by all household members inside and outside of the home, excluding foods purchased and eaten outside of the home (Swindale & Bilinsky, 2006). Dietary data were collected using a qualitative, 24‐h open recall method. The household dietary data of the previous day were provided by the primary person responsible for food preparation and purposes, most often the primary female. The respondent was asked to mention, in order starting from the morning, all the foods and drinks consumed in the last 24 h. The respondent was then probed on ingredients in mixed dishes and snacks. Data collectors checked off the food groups that were mentioned and followed up on food groups that were blank at the end of the recall. A score of 0–12 was then constructed based on the following food groups: (1) cereals, (2) roots and tubers, (3) vegetables, (4) fruits, (5) meat, poultry, offal, (6) eggs, (7) fish and seafood, (8) pulses, legumes and nuts, (9) milk and milk products, (10) oils/fats, (11) sugar/honey and (12) miscellaneous (FAO, 2011; Swindale & Bilinsky, 2006).

Women's dietary diversity was also collected using the Women's Dietary Diversity Score (WDDS) (FAO, 2011). WDDS reflects the probability of micronutrient adequacy of the diet, putting more emphasis on micronutrient intake than on economic access to food (FAO, 2011). Dietary data were collected using a qualitative, 24‐h open recall method, similar to the HDDS (FAO, 2011). A score of 0–9 was constructed based on the following food groups: (1) starchy staples, (2) dark green leafy vegetables, (3) other vitamin‐A rich fruit and vegetables, (4) other fruits and vegetables, (5) organ meat, (6) meat and fish, (7) eggs, (8) legumes, nuts and seeds and (9) milk and milk products (Arimond & Ruel, 2004; FAO, 2011). The women's dietary diversity module was adapted in the Tanzania endline to allow for the construction of the newer Minimum Dietary Diversity for Women (MDD‐W) indicator (FAO and FHI 360, 2016), but we present findings for WDDS because we have panel data for this indicator. We discuss the implications of this later in the paper.

### 2.8 Children's anthropometric assessment

Anthropometric data included measurements of height and weight for all children from 6 to 59 months undertaken during home visits. Recumbent length of children aged <2 years and standing height of children aged >2 years were measured to the nearest 0.1 cm by using portable length/height boards, and weight was measured to the nearest 0.1 kg with the use of electronic scales. Linear growth was examined by height for age *z*‐scores (HAZ) and prevalence of stunting. Following World Health Organization's (WHO) recommendations, children with values outside the biologically plausible range with HAZ <−6 or >6 or WHZ <−5 or >5 were excluded from analysis, since these values are most likely the result of measurement errors (WHO Multicentre Growth Reference Study Group, 2006). A child is stunted if her HAZ is below minus two standard deviations (SD) from the median of the WHO reference population. Similarly, a wasted child has a WHZ below minus two SD from the median of the WHO reference population.

### 2.9 Statistical analysis

The analysis uses a panel fixed effects estimation to examine the impact of irrigation on HDDS (a proxy measure of household food access), WDDS and children's nutrition indicators including WHZ, wasting, HAZ and stunting. When at least two periods of data are available, the panel fixed effects approach uses transformation of equations over time to eliminate the effect of any observed or unobserved time‐invariant variable on the outcome (dependent) variable. The transformation of equations uses either first differencing of equations over time or subtracts the average of the equations over time from the original equation. The model for wasting was not estimated for Tanzania due to small sample size (only 2.2% of children under five in the sample were wasted). All data were imported into Stata version 15 (StataCorp) for statistical analysis. The regression uses a linear probability model, instead of a logit model, due to the limited variation in irrigation status over time. Panel fixed‐effects logistic models drop observations whose values do not vary over time.

All household and women‐level regressions control for the effects of time‐varying socioeconomic characteristics, including access to irrigation, a dummy variable for the survey round, size of land ownership, number of adults in the household, ownership of cows, ownership of goats and sheep, ownership of chickens, remittances, self‐employment income, whether the adult woman had a formal education, reported shocks such as storms, drought and floods, crop damage from pests and disease, and idiosyncratic shocks, such as death and illness of a household member. Irrigation may have different impacts during a drought as well as across different survey rounds, for instance, due to differences in the price farmers receive for their produce during that season. Thus, we included the interaction of the irrigation variable with the occurrence of drought and with the different survey rounds to capture the differential impact of irrigation across different survey periods and weather conditions.

Child‐level regressions control for all the above household‐level socioeconomic characteristics as well as child‐specific variables, including age in months, number of siblings under 5 years of age, whether the child was sick in the 2 weeks preceding the survey, and whether the child was exclusively breast‐fed for the first 6 months.

The panel fixed effects in all the household, women and child‐specific estimations control for factors that can affect the outcome variables but that can reasonably be expected not to have changed over the 2 years between the survey periods. Thus, time‐invariant village and household‐level influences are effectively controlled for, facilitating a credible identification of the effect of irrigation (and other time‐varying explanatory variables) on the dietary and nutritional outcomes of interest. Standard errors are clustered by village as dietary and nutritional outcomes of households and individuals in the same village may be correlated due to their sharing of, for example, similar market, infrastructure and other constraints.

## 3 RESULTS

Table 1 provides the summary statistics of household‐level outcome and explanatory variables in Ethiopia and Tanzania. The results show that irrigators in Ethiopia have higher HDDS, own more land, own more chickens and have more children under 5 years of age compared to nonirrigators (Table 1, Columns 1–3). On the other hand, irrigators in Ethiopia are less likely to report shocks such as drought, storm and flood, own slightly less number of small ruminants, such as goats and sheep, receive fewer gifts and remittances, and report fewer idiosyncratic shocks, such as illness and death in the family (Table 1, columns 1–3).

**Table 1:** Descriptive statistics of household‐level outcome and explanatory variables in Ethiopia and Tanzania

	Ethiopia			Tanzania		
	(1)	(2)	(3)	(4)	(5)	(6)
	Nonirrigators	Irrigators	Difference	Nonirrigators	Irrigators	Difference
HDDS (0–12)	5.62	5.864	−0.244***	5.72	6.05	−0.321***
WDDS (0–9)	3.46	3.431	0.025	3.85	4.23	−0.375***
Drought occurred (0/1)^a^	0.105	0.059	0.046***	0.22	0.181	0.040
Total land owned (ha)	1.72	2.041	−0.322***	3.96	3.32	0.635**
Owns cows (0/1)	0.85	0.844	0.002	0.043	0.038	0.005
Owns goats or sheep (0/1)	0.596	0.537	0.060*	0.119	0.059	0.060***
Owns chickens or birds (0/1)	0.739	0.812	−0.073***	0.660	0.630	0.030
Insect/disease damage (0/1)	0.083	0.072	0.011	0.136	0.155	−0.019
Household size (number)	6.19	6.202	−0.014	4.86	4.99	−0.125
# of children under 5	0.594	0.699	−0.105**	0.579	0.641	−0.062
Woman's education (years)	0.325	0.319	0.007	2.32	2.93	−0.611***
Remittances (0/1)	0.121	0.074	0.047**	0.138	0.123	0.015
Employment income (0/1)	0.219	0.189	0.030	0.399	0.433	−0.034
Gifts (0/1)	0.107	0.038	0.069***	0.066	0.083	−0.016
Illness/death in family (0/1)	0.190	0.149	0.041*	0.284	0.313	−0.029
Storm/flood	0.093	0.043	0.049***	0.180	0.181	−0.001
Observations	421	531		589	426	

Abbreviations: HDDS, Household Dietary Diversity Score; WDDS, Women's Dietary Diversity Score.

^a^ 0/1 refers to responses where No = 0 and Yes = 1.

*p < 0.1; **p < 0.05; ***p < 0.01.

In Tanzania, irrigators have higher HDDS and WDDS but own less land and fewer goats and sheep (Table 1, columns 4–6). Women in irrigating households in Tanzania have more years of education compared to those in nonirrigating households (Table 1, columns 4–6).

Table 2 shows summary statistics of children‐related outcome and explanatory variables in Ethiopia and Tanzania. In Ethiopia, the descriptive analysis shows no statistically significant differences for nutritional and other related variables by irrigation status (Table 2, columns 1–3). In Tanzania, WHZ‐scores are higher for children in irrigating households compared to those in nonirrigating households. In addition, the sample of irrigated households contains a greater number of children aged 12–24 months, but a smaller number of children aged 24–59 months compared to the sample of nonirrigating households.

**Table 2:** Descriptive statistics of children‐related outcome and explanatory variables in Ethiopia and Tanzania

	Ethiopia			Tanzania		
	(1)	(2)	(3)	(4)	(5)	(6)
	Nonirrigators	Irrigating households	Differences	Nonirrigators	Irrigators	Difference
WHZ‐score	−0.151	−0.029	−0.122	0.065	0.220	−0.155*
Wasting (0/1)^a^	0.062	0.083	−0.0215	0.022	0.022	0.001
HAZ‐score	−1.647	−1.567	−0.0804	−1.274	−1.250	−0.024
Stunting (0/1)	0.411	0.399	0.0119	0.266	0.264	0.002
6–12 months old^b^	0.212	0.187	0.0250	0.149	0.169	−0.020
12–24 months old^b^	0.182	0.234	−0.0521	0.153	0.221	−0.068*
24–59 months old^b^	0.606	0.579	0.0271	0.698	0.610	0.087**
Sick the previous 2 weeks (0/1)	0.357	0.316	0.0405	0.401	0.430	−0.030
Exclusive breastfeeding (0/1)	0.504	0.567	−0.0622	0.365	0.383	−0.019
Observations	573		573	499		499

*Note*: (0/1) refers to a dummy variable where yes = 1 and no = 0.

Abbreviations: WHZ, weight‐for‐height; HAZ, height‐for‐age.

^a^ 0/1 refers to responses where No = 0 and Yes = 1.

^b^ Refers to number of children in that age group in the household.

*p < 0.1; **p < 0.05; ***p < 0.01.

The presence of statistically significant differences in several factors between irrigators and nonirrigators implies the need to control for such confounding factors as one explores the linkage between irrigation and nutritional status. Even when the potential explanatory variables do not differ by irrigation status, there could be unobserved effects that determine access to irrigation (such as availability of groundwater on farmers' plots, distance to surface water bodies, access to financial resources or farmers' preference and prior exposure to irrigation) that can complicate the analysis of irrigation‐nutrition linkages using only descriptive statistics such as *t*‐tests. Thus, in what follows, we present results from a panel fixed effects estimator that controls for both time‐varying observed potential explanatory variables and time‐invariant confounders (observed or unobserved) that could be correlated with access to irrigation status.

Since our focus is on exploring linkages between use of small‐scale irrigation and nutritional outcomes of households, women and children, we only discuss a subset of the marginal effects of the estimates pertaining to access to irrigation and its interactions with drought occurrence and survey rounds. Parameter estimates from the full model are available online as Supporting Information.

### 3.1 Household and WDDS by irrigation status

In Tables 3 and 4, we present the average marginal effects of irrigation on HDDS and WDDS, and its potentially heterogeneous effects across survey rounds and drought shocks in Ethiopia and Tanzania. Table 3 shows that, overall, HDDS and WDDS were lower in the 2017 survey round compared to the 2015 survey round in Ethiopia.

**Table 3:** Average marginal effects of access to irrigation on WDDS and HDDS in Ethiopia

	(1)	(2)
	HDDS	WDDS
Nonirrigators	Base/reference group	Base/reference group
Irrigators, (1 = yes, 0 = no)	0.113	0.119
	(0.149)	(0.158)
Drought occurred, (1 = yes, 0 = no)	0.232	−0.251
	(0.221)	(0.212)
February to April 2017, (1 = yes, 0 = no) (Dec 2014 as reference season)	−0.246***	−0.236*
	(0.087)	(0.122)
Irrigation–season interactions		
December 2014	0.279	−0.147
	(0.225)	(0.203)
Feb to April 2017	−0.020	0.333*
	(0.152)	(0.177)
Irrigation–drought interactions		
No drought	0.111	0.102
	(0.152)	(0.167)
Drought	0.143	0.308*
	(0.284)	(0.172)
Observations	950	950

*Note*: Standard errors in parentheses.

Abbreviations: HDDS, Household Dietary Diversity Score; WDDS, Women's Dietary Diversity Score.

*p < 0.1; **p < 0.05; ***p < 0.01.

The results in column 2 of Table 3 show that women in irrigating households in Ethiopia have higher WDDS during the second round of the survey (February to April 2017) and among households who reported drought shocks (in the 5 years before the baseline survey or in the 2 years between the two surveys). Women in irrigating households had more diversified diets during the main fasting season, with WDDS higher by 0.33, which amounts to 9.6% of the average WDDS of nonirrigators in Ethiopia. Among households who reported facing drought at least once in the 5 years before the baseline survey or the 2 years between the two surveys in Ethiopia, WDDS is higher by 0.31 for women in irrigating households compared to those in nonirrigating households (a difference equivalent to 9% of the average WDDS score of 3.44).

In Tanzania, women in irrigating households had, on average, a higher WDDS of 0.36 compared to those in nonirrigating households—a difference equivalent to 9% of the average WDDS score of 4 (Table 4). Among households who did not report a drought in Tanzania, women in irrigating households have more diversified diets (WDDS higher by 0.29) compared to women in nonirrigating households (Table 4). Among households who reported drought during the reference period, women in irrigating households have a WDDS higher by 0.64 compared to women in nonirrigating households (Table 4). In Tanzania, irrigation also has a positive and statistically significant impact on HDDS (by about 18%) among households who faced drought during the reference period.

**Table 4:** Average marginal effects of access to irrigation on WDDS and HDDS in Tanzania

	(1)	(2)
	HDDS	WDDS
Nonirrigators	Base/reference group	Base\reference group
Irrigators, (1 = yes, 0 = no)	0.174	0.363**
	(0.273)	(0.171)
Drought occurred, (1 = yes, 0 = no)	0.437*	−0.279*
	(0.231)	(0.145)
July 2017 (1 = yes, 0 = no) (July 2015 as reference season)	0.645***	−0.030
	(0.234)	(0.202)
Irrigation–season interactions		
July 2015	0.429	0.431***
	(0.323)	(0.159)
July 2017	−0.023	0.304
	(0.262)	(0.216)
Irrigation–drought interactions		
No drought	−0.051	0.291*
	(0.276)	(0.173)
Drought	1.05**	0.637**
	(0.536)	(0.350)
Observations	972	972

*Note*: Standard errors in parentheses.

Abbreviations: HDDS, Household Dietary Diversity Score; WDDS, Women's Dietary Diversity Score.

*p < 0.1; **p < 0.05; ***p < 0.01.

### 3.2 Child level nutrition outcomes

Tables 5 and 6 show the overall effect of irrigation on children's nutritional outcomes in Ethiopia and Tanzania, as well as heterogeneous effects by survey round and exposure to drought. Table 5 shows that, overall, children in irrigating households in Ethiopia have WHZ‐scores that are 0.87 SDs higher, on average, than the WHZ of children in nonirrigating households. The positive effect of irrigation on WHZ is much stronger in the second round of the survey (February to April 2017), where children in irrigating households have a 1.1 SD higher WHZ than children in nonirrigating households.

**Table 5:** Average marginal effects of parent's access to irrigation on children's nutritional status in Ethiopia

	(1)	(2)	(3)	(4)
	WHZ	Wasting	HAZ	Stunting
Nonirrigators	Base/reference group	Base/reference group	Base/reference group	Base/reference group
Irrigators (1 = yes, 0 = no)	0.873*	0.037	−0.322	−0.015
	(0.448)	(0.074)	(0.451)	(0.153)
Drought occurred, (1 = yes, 0 = no)	−0.003	0.155**	−0.173	−0.046
	(0.872)	(0.070)	(0.289)	(0.074)
Feb to April 2017, (1 = yes, 0 = no) (Dec 2014 as reference season)	−0.213	−0.111	1.26***	−0.239**
	(0.458)	(0.071)	(0.309)	(0.117)
Irrigation–season interactions				
December 2014	0.524	0.056	0.472	−0.024
	(0.521)	(0.090)	(0.567)	(0.165)
Feb to April 2017	1.09**	0.026	−0.798	−0.010
	(0.529)	(0.073)	(0.442)	(0.167)
Irrigation–drought interactions				
No drought	0.803	0.075	−0.266	−0.061
	(0.507)	(0.074)	(0.474)	(0.172)
Drought	1.34	−0.218	−0.715	0.312
	(0.981)	(0.148)	(0.749)	(0.253)
Observations	461	461	461	461

*Note*: standard errors in parentheses.

Abbreviations: WHZ, weight‐for‐height; HAZ, height‐for‐age.

*p < 0.1; **p < 0.05; ***p < 0.01.

Table 5 also shows that drought exacerbates wasting of children under 5 years of age (Table 5, column 2). Columns 3 and 4 of Table 5 show that HAZ‐scores have improved, and stunting has declined in the Ethiopia sample between the two survey periods, consistent with other reports of HAZ improvements and stunting reduction in Ethiopia over time. According to data from the Ethiopia Demographic and Health Surveys, stunting rates among children younger than 5 years were 51% in 2005, subsequently declining to 44% and 38% in 2011 and 2016. The negative sign on the interaction between irrigation status and the second round of the survey on HAZ (Table 5, column 3) suggests that the change over the 2 years is stronger for children in nonirrigating households and indicates narrowing of differences in stunting between children in irrigating and nonirrigating households over time. This effect might be due to interventions unrelated to irrigation. This result, coupled with the positive sign of the marginal effect of the interaction between irrigation status and the second round of the survey on WHZ (Table 5, column 1), implies that irrigation affects more acute nutritional deficiencies (as captured by WHZ and wasting) but not long‐term growth (as captured by HAZ and stunting).

In Tanzania, irrigation leads to higher WHZ‐scores of children under 5 years of age among households who reported experiencing drought at least once in the previous 5 years. Children in these households have 0.62 SDs higher WHZ than those in nonirrigating households (column 1 of Table 6). In between the two survey rounds in 2015 and 2017, results show an improvement in HAZ‐scores and a corresponding decline in stunting among children in the Tanzanian sample (columns 2 and 3 of Table 6), similar to Ethiopia.

**Table 6:** Average marginal effects of parent's access to irrigation on children's nutritional status in Tanzania

	(1)	(2)	(3)
	WHZ	HAZ	Stunting
Nonirrigators	Base/reference group	Base/reference group	Base/reference group
Irrigators (1 = yes, 0 = no)	0.203	−0.108	−0.207
	(0.414)	(0.417)	(0.192)
Drought occurred, (1 = yes, 0 = no)	0.197	0.599**	−0.027
	(0.215)	(0.264)	(0.129)
July 2017, (1 = yes, 0 = no) (July 2015 as reference season)	0.311	1.17*	−0.678***
	(0.454)	(0.626)	(0.211)
Irrigation–season interactions			
July 2015	0.459	−0.564	−0.054
	(0.549)	(0.353)	(0.180)
July 2017	0.013	0.221	−0.317
	(0.332)	(0.594)	(0.207)
Irrigation–drought interactions			
No drought	0.111	−0.077	−0.234
	(0.491)	(0.523)	(0.229)
Drought	0.616*	−0.252	−0.080
	(0.322)	(0.532)	(0.175)
Observations	453	453	453

*Note*: Wasting was not estimable in Tanzania due to insufficient observations with wasting equal to 1. Standard errors in parentheses.

Abbreviations: WHZ, weight‐for‐height; HAZ, height‐for‐age.

*p < 0.1; **p < 0.05; ***p < 0.01.

## 4 DISCUSSION

This study provides evidence to support the positive influence of irrigation on food access and nutrition in rural areas of developing countries, where rainfed agriculture is the main source of livelihood, and climatic shocks are among the main risks to agricultural production and well‐being.

Climate and idiosyncratic shocks are shown to have a negative impact on dietary diversity and child nutrition in both countries. Access to irrigation tends to increase household and women's resilience to drought shocks. Among households who reported having experienced drought, the strong and statistically significant effect of irrigation on HDDS and WDDS in Tanzania and on WDDS in Ethiopia suggest that irrigation increases households' resilience to drought in a manner that can counteract the negative effects of droughts on the nutritional status of households. In a subsistence setting where most of the food consumed in the household comes from own production, exposure to drought and subsequent reduction in production will directly impact household food consumption. Even when farmers sell most of their produce, the income shock from drought will directly affect food consumption. There are at least two possible reasons for the decrease in HDDS and WDDS in 2017. First, the 2017 survey was undertaken between February and April, which is the main lent season in which followers of the Ethiopian Orthodox Church (82% of households in the sample) abstain from consumption of all livestock products and reduce meal frequency in the 55 days before Easter. Though there is another fasting period in December as well, the level of observance is not as strict as the lent fasting season. Second, unlike the December 2014 survey round, the February to April survey period in 2017 is further away from the main harvest season of December.

The study documents differential nutritional status of children under five in households with and without irrigation. Children in irrigating households in Ethiopia have WHZ‐scores that are 0.87 SDs higher, on average, than WHZ‐scores of children in nonirrigating households. In Tanzania, irrigation leads to higher WHZ‐scores of children under 5 years of age among households who reported facing drought.

In Ethiopia, the positive effect of irrigation on WHZ‐scores is not large enough to be translated into reducing wasting (as a 0/1 indicator variable). That is, while irrigation improves WHZ‐scores, the change is not large enough to change children's nutritional status from being wasted to not being wasted. In both Ethiopia and Tanzania, there are no statistically significant relationships between irrigation on the one hand and HAZ‐scores and stunting on the other. This is likely because stunting or chronic malnutrition is a measure of linear growth retardation and cumulative growth deficit in children due to undernutrition and illnesses that deprive a fetus and child of required macro‐ and micronutrients. It is largely an irreversible outcome capturing 'past and future predictions' unveiling the growth environment of individuals as well as children's potential to fulfil their development and economic potential (Leroy & Frongillo, 2019). As such, access to irrigation may not reverse the harm that has already been done.

On the other hand, wasting, or acute malnutrition, indicates an acute state of deficiency due to recent drastic weight loss often due to a sudden reduction in food intake or an illness both of which can result from drought. Drought may also increase the risk of diarrhoea and decreased body weight if it forces households to use less protected and safe water sources. While more children are stunted than wasted globally (and in Ethiopia), wasted children face higher risk of death than stunted children absent necessary treatments (Black et al., 2008). Previous research has shown higher wasting among children affected by drought including in Ethiopia (Delbiso et al., 2017). The stronger effect of irrigation on WHZ in the 2017 survey round could be due to the longer period of exposure to irrigation technologies and their benefits by this survey round compared to that of 2014.

This paper has some limitations. First, access to small‐scale irrigation is not random, and hence unobserved individual heterogeneities that may correlate with having access to irrigation may also influence women's and household diet diversities. However, we adjust for potential bias in the regression by exploiting the two rounds of data from the same households that enable us net‐out the effect of such individual heterogeneities if they can reasonably be assumed to be invariant over the 2 years between the surveys. Although the sample size in both Tanzania and Ethiopia were large enough to detect the impact of irrigation on nutrition and dietary outcomes, we were unable to assess the effects of specific irrigation technologies due to the low sample size of each technology. Moreover, in Ethiopia, the endline survey was conducted in a different season than the baseline. This was due to security and logistical issues related to demonstrations that took place in the survey areas during December 2016 and January 2017. Data collection thus occurred between February and April 2017 and, as discussed previously, coincided with lent, a period in which many observers reduce the number of meals consumed in a day and adopt a vegan diet. This may have impacted both the HDDS and WDDS of the endline data, making it difficult to capture the full effects of irrigation on dietary outcomes. The strengths of this paper include the panel data set at the household and individual level that allows for attributing causal relationships from an observational data and the rich nature of the data in its coverage of both agriculture and nutrition‐related information of farm households. Moreover, by including drought as an explanatory variable in the analysis and how it interacts with access to irrigation to affect women's and households' diet, we were able to capture the conditions in which irrigation acts as a buffer for dietary and nutritional outcomes.

In conclusion, this study has shown that irrigation has a strong effect on households' economic access to food and on nutritional outcomes of women and children, effects that are stronger among households that experienced a drought shock. As such, irrigation should be promoted on its merit to improve nutrition, in addition to its potential for higher yields, incomes and employment. This is especially important for areas prone to recurring and severe drought and considering expected climatic changes that will increase the frequency, intensity and duration of climatic shocks. Expansion of small‐scale irrigation can enable Ethiopia and Tanzania to fully realize their potential and capacity to produce enough not only for local consumption but also for export. While the Ethiopian and Tanzanian governments have made irrigation expansion a policy priority in recent years, including by passing fiscal and nonfiscal legislations, more work is needed to alleviate institutional, infrastructural and market constraints that make irrigation technologies effectively inaccessible to millions of smallholders.

## AUTHOR CONTRIBUTIONS

The author contributions were as follows: Claudia Ringler, Dawit K. Mekonnen and Elizabeth Bryan designed the study; Dawit K. Mekonnen, Elizabeth Bryan and Jowel Choufani led the data collection activities; Jowel Choufani provided guidance on preparation of the nutrition indicators; Dawit K. Mekonnen led the data analysis and write up; Beliyou Haile worked on the descriptive analysis; Claudia Ringler provided overall management of the project; Dawit K. Mekonnen had the responsibility for submitting this article for publication; and all authors contributed to interpreting the results in this study, had full access to the data, and read and approved the final version of the manuscript.

## CONFLICT OF INTEREST

The authors declare no conflict of interest.

## Supporting information

Supporting information.

Supporting information.

## Data Availability

The first round of the data is publicly available here on Harvard data verse https://dataverse.harvard.edu/dataset.xhtml?persistentId=doi:10.7910/DVN/DH1O3J. The second round of the data will also be made public soon on the same website. The documentation of the second round of the data is currently being finalized.
