# Supplementary material for: Irrigation improves weight‐for‐height z‐scores of children under five, and Women's and Household Dietary Diversity Scores in Ethiopia and Tanzania
Source: Matern Child Nutr. 2022 Jun 24;18(4):e13395. doi: 10.1111/mcn.13395 (PMC9480923; doi:10.1111/mcn.13395)
Supplement: Supplementary file 1 — Supporting information. [file MCN-18-e13395-s002.docx]

# **Appendix A Study sample**

The table below summarizes the timing and coverage of two rounds of surveys conducted in Ethiopia and Tanzania.

Table A Summary of Study Sample

| Survey | Ethiopia | Tanzania |
| --- | --- | --- |
| Round 1–  timing | November 14^th^ - December 26^th^ 2014 | June 24th – July 11th, 2015 |
| Round 1–sample | 15 villages  439 households  Bahir Dar Zuria and Dangla districts in Amhara region, Adami Tulu district in Oromia region, and Lemu districts in SNNPR region | 14 villages  451 households  Kilosa and Mvomero districts of Morogoro region |
| Round 2–  timing | February 20th – April 12, 2017 | June 26 to July 26, 2017 |
| Round 2–sample | 15 villages  549 households (439 households from baseline plus additional 100 SIPS households) | 17 villages  540 households  Kilosa, Mvomero, and Babati districts |

# **Appendix B: Sampling Details**

Sampling details for Ethiopia and Tanzania surveys can be found in (Mekonnen et al. 2019), which we have reproduced here with authors’ permission for easy access.

***Ethiopia:***

The sample for the household survey was drawn from the 4 woredas (districts) in which IWMI/ILRI interventions on small-scale irrigation took place: Dangla and Bahir-Dar Zuria in Amhara Region, Lemo in SNNPR Region, and Adami Tulu in Oromia Region. For each of these woredas, we obtained a list of kebeles (villages) with data on population size (using CSA data) and suitability for irrigation based on the ex-ante suitability analysis conducted for the Agricultural Water Management (AWM) Solutions project. A document describing the methodology for small-scale irrigation analysis, which explains how the average suitability scores were calculated for all rural kebeles in these 4 woredas, is included in the appendix.

In addition to the 4 intervention kebeles (one in each woreda), we randomly selected 11 additional kebeles from within the 4 woredas in the following way. In each woreda, we first maintained the kebeles with an irrigation suitability score of 30 and above to ensure that the sampling frame of villages from which we randomly select the sampled villages will have relatively similar ex-ante suitability for irrigation as the four intervention kebeles. Then we used the total population size (number of households) per kebele as a weight to determine the number of kebeles to be randomly selected from each woreda. This resulted in the following breakdown:

| Woreda | No. of villages randomly selected | Total number of villages (including intervention villages) |
| --- | --- | --- |
| Adami Tulu | 1 | 2 |
| Bahir Dar Zuria | 6 | 7 |
| Dangla | 2 | 3 |
| Lemo | 2 | 3 |
| Total | 11 | 15 |

In other words, the number of kebeles selected per woreda depended on the size of the population living in high potential areas within that woreda. Within the woreda, the intervention kebeles, (which were not randomly selected) were included the sample. The remaining kebeles were drawn randomly with probability proportional to size. This resulted in the following sample of kebeles:

| Woredas/kebeles | | Mean suitability score | No. of households |
| --- | --- | --- | --- |
| Adami Tulu (2) | |  |  |
| 1 | Edo Gojola | 67.48 | 603 |
| 2 | Bochesa* | 58.62 | 474 |
| Bahir Dar Zuria (7) | |  |  |
| 1 | Yegoma Huletu | 58.89 | 1,997 |
| 2 | Robit* | 52.66 | 1,916 |
| 3 | Wejir Welda Menta | 50.94 | 1,166 |
| 4 | Tis Abay Town | 49.82 | 4,369 |
| 5 | Meshenti Town | 47.17 | 1,101 |
| 6 | Wegeligo | 44.88 | 970 |
| 7 | Gomibat Aba Gerima | 40.00 | 1,235 |
| Dangla (3) | |  |  |
| 1 | Gumbri Abela Akana | 44.77 | 1,236 |
| 2 | Ligaba | 43.12 | 667 |
| 3 | Dangishta** |  |  |
| Lemo (3) | |  |  |
| 1 | Ajo Teasa | 42.09 | 565 |
| 2 | Digba | 36.63 | 738 |
| 3 | Upper Gana* | 34.01 | 609 |
| Note: *intervention site; **The kebele selected for the IWMI/ILRI intervention in Dangla (Dangishta kebele) did not appear on our GIS shape file or in the population data provided by CSA, so we do not have these data for this site. | | | |

Within each of the chosen kebeles, we obtained a list of households from the local extension office with an indication of whether the household uses irrigation or not. We randomly selected 10 households from the list of irrigators and 10 households from the list of non-irrigators for a total of 20 households per kebele. For the purposes of this data paper, irrigators are defined as households who irrigated at least one plot in either the main rainy season (Meher) or the dry small-showers season (Belg). In the few cases where the information on households’ irrigation status provided by the extension office differs from what we gather from the household survey, we used households’ response to determine their irrigation status. Table 1 shows the distribution of the sample for Ethiopia.

Table 1: Sample Distribution for Ethiopia

| Ethiopia | | | | | |
| --- | --- | --- | --- | --- | --- |
| Region | Zone | Woreda | Kebele | Total HHs | Irrigators (%) |
| Amhara | Awi | Dangla | Dangishta | 44 | 54.55 |
| Amhara | Awi | Dangla | Gumbri Abela Akana | 20 | 50.00 |
| Amhara | Awi | Dangla | Ligaba | 20 | 50.00 |
| Amhara | West Gojjam | Bahir Dar Zuria | Gomibat Aba Gerima | 20 | 100.00 |
| Amhara | West Gojjam | Bahir Dar Zuria | Meshenti Town | 21 | 52.38 |
| Amhara | West Gojjam | Bahir Dar Zuria | Robit | 64 | 93.75 |
| Amhara | West Gojjam | Bahir Dar Zuria | Tis Abay Town | 20 | 45.00 |
| Amhara | West Gojjam | Bahir Dar Zuria | W/W/ Menta | 19 | 47.39 |
| Amhara | West Gojjam | Bahir Dar Zuria | Wegeligo | 20 | 50.00 |
| Amhara | West Gojjam | Bahir Dar Zuria | Yegoma Huletu | 19 | 52.63 |
| Oromia | East Shoa | Adami Tulu | Bochessa | 45 | 48.89 |
| Oromia | East Shoa | Adami Tulu | Edo Gojola | 20 | 50.00 |
| SNNPR | Hadiya | Lemu | Ajo Teasa | 20 | 50.00 |
| SNNPR | Hadiya | Lemu | DigbaTachignawe Kode Duna | 20 | 45.00 |
| SNNPR | Hadiya | Lemu | Upper Gana | 65 | 38.46 |
|  |  |  | Total | 437 | 56.98 |
| Note: HHs = households; 2 HH missing for Ethiopia | | | | | |

***Tanzania:***

The sample for the household survey was drawn from the 2 districts in which IWMI interventions on small-scale irrigation took place: Kilosa and Mvomero districts in Morogoro region. For both of these districts, we obtained a list of villages with data on population size (using the 2012 Population census data from Tanzania National Bureau of Statistics) and suitability for irrigation (at the ward level) based on the ex-ante suitability analysis conducted for the AWM Solutions project and later refined through the World Bank drylands study. A document describing the methodology for small scale irrigation analysis, which explains how the average suitability scores were calculated for all rural wards in these 2 districts, is included in the appendix.

In addition to the 2 intervention villages (one in each district), we randomly selected 12 additional villages from within the 2 districts in the following way. We first determined the number of wards in the district with an average irrigation suitability score of 30 or greater. We then calculated the total population size living within high irrigation potential wards in each district. We determined the number of villages to sample from each district based on the proportion of households living in areas with a high suitability score. This resulted in the following breakdown:

| District | No. of villages randomly selected | Total number of villages (including intervention villages) |
| --- | --- | --- |
| Kilosa | 7 | 8 |
| Mvomero | 5 | 6 |
| Total | 12 | 14 |

In other words, the number of villages selected per district depended on the size of the population living in high potential areas within that district. Within the district, the selected number of villages was drawn randomly with probability proportional to population size, with the exception of villages in which the IWMI/ILRI interventions would place. The intervention sites were not selected at random but based on scoping work done by IWMI/ILRI. Adding the two intervention sites brings the total number of villages in the sample to 14. This resulted in the following sample of villages:

| Districts/villages | | Mean suitability score | No. of households |
| --- | --- | --- | --- |
| Kilosa (8) | |  |  |
| 1 | Chanzulu | 35.87 | 3,617 |
| 2 | Ilonga | 35.87 | 6,039 |
| 3 | Kimamba 'A' | 67.25 | 6,076 |
| 4 | Kibaoni | 52.27 | 2,218 |
| 5 | Kondoa | 52.27 | 1,564 |
| 6 | Madudumizi | 47.24 | 3,882 |
| 7 | Zombolumbo | 47.24 | 3,068 |
| 8 | Rudewa Mbuyuni* | 12.49 | 3,627 |
| Mvomero (6) | |  |  |
| 1 | Magali | 41.50 | 2,263 |
| 2 | Mangae | 41.50 | 2,543 |
| 3 | Sangasanga | 33.85 | 1,716 |
| 4 | Vikenge | 33.85 | 2,175 |
| 5 | Tangeni | 33.85 | 5,386 |
| 6 | Mkindo* | 27.06 | 6448 |
| Note: *intervention site | | | |

Within each of these villages, we obtained a list of households from the local extension office with an indication of whether the household uses irrigation or not. We randomly selected 14 households from the list of irrigators and 14 households from the list of non-irrigators for a total of 28 households per village. For the purposes of this data paper, irrigators are defined as household who irrigated at least one plot in either the main rainy season or the dry season. While there is overlap between the information provided by the local extension office, some households are coded differently. Table 2 shows the distribution of the sample for Tanzania.

Table 2: Sample Distribution for Tanzania

| **Tanzania** | | | | | |
| --- | --- | --- | --- | --- | --- |
|  |  | **District** | **Village** | **Total HHs** | **Irrigators (%)** |
|  |  | Kilosa | Chanzulu | 28 | 60.71 |
|  |  | Kilosa | Ilonga | 28 | 60.71 |
|  |  | Kilosa | Kibaoni | 28 | 39.29 |
|  |  | Kilosa | Kimamba 'A' | 28 | 39.29 |
|  |  | Kilosa | Kondoa | 29 | 48.28 |
|  |  | Kilosa | Madudumizi | 28 | 35.71 |
|  |  | Kilosa | Rudewa Mbuyuni* | 58 | 46.55 |
|  |  | Kilosa | Zombolumbo | 28 | 60.71 |
|  |  | Mvomero | Magali | 28 | 50.00 |
|  |  | Mvomero | Mangae | 28 | 50.00 |
|  |  | Mvomero | Mkindo* | 56 | 69.64 |
|  |  | Mvomero | Sangasanga | 28 | 28.57 |
|  |  | Mvomero | Tangeni | 28 | 50.00 |
|  |  | Mvomero | Vikenge | 28 | 50.00 |
|  |  |  | **Total** | **451** | **50.33** |
| *Note:* HHs = households | | | | | |

# **Appendix C Irrigation suitability**

Table C1 Criteria and data for ex-ante suitability analysis

| Criteria | Data set | Explanation |
| --- | --- | --- |
| Topography (slope) | SRTM (Shuttle Radar Topography Mission) elevation data | SSI tends to occur in area with gentle slope; the slope data layer is derived from SRTM elevation data |
| Groundwater accessibility^2^ | BGS (British Geological Survey) quantitative groundwater map for Africa | The BGS groundwater map provides quantitative estimates of depth to groundwater, which can be used as an indicator of accessibility of groundwater |
| Distance to perennial surface water | Global Lakes and Wetlands  Database (Lehner and Doll,  2004), the V-Map Perennial  Streamlines dataset (National  Imagery and Mapping  Agency 1997) |  |
| Distance to main river course | Streamlines dataset (National  Imagery and Mapping Agency 1997) and Dam list of this project (various  sources) | For large scale irrigation (LSI) only. Water access point for LSI are normally from the main river where a dam controls the water flow  and availability |
| Proximity to existing irrigation | FAO Irrigation Scheme Map (Sirte Africa Large Irrigation Map) | Markets for inputs and outputs are already developed, and farmers have seen irrigation, are familiar with it, know the technology, and are enthusiastic about getting in on the action. |
| Market access | Nelson (2008) travel time | Market access is included as a criterion because adoption of irrigation relies on markets both for equipment/facility maintenance and sales of crop products. |

1. Protected and area/existing irrigated areas^1^ will be excluded from environmental suitability analysis.
2. Not applicable for LSI.

To measure irrigation potential, we divide the criteria parameters summarized above into 3 classes and use a linear interpolation within the classes to calculate the scores. Such classification is similar to a stepwise function that gives us the flexibility to adjust the threshold values after consulting with experts and stakeholders. The overall rating of the irrigation suitability is the average of total scores for all applicable criteria. Since both ground and surface water provides the same water resource to irrigation, we take the larger score of these two in calculating overall suitability as shown below.


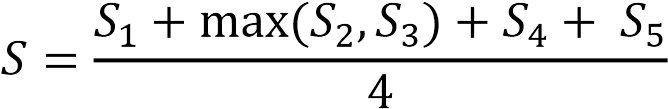


where S is irrigation suitability score, S_1,_ S_2,_ S_3,_ S_4,_ and S_5_ are the scores for slope, surface water access, ground water access, ground distance to existing large-scale irrigation schemes, and market access, respectively.

The suitability analysis is done on a 0.5km x 0.5km grid and we aggregate the finer resolution analysis into a 10 km x10 km grid (SPAM grid) for our Step 2 analysis. The suitability score is the average of those pixels within the coarser grid. We then use the suitability score as a percent of the pixel suitable for irrigation, so that the total suitable irrigation area would be the weighted sum of pixel area and suitability score (0 to 1). This would maintain the suitability functions developed for the ex-ante analysis without filtering the results through another processing step summarized in Table C2.

Table C2 Scoring scheme used for irrigation suitability analysis

|  |  | Unit | Range | Class 1 | Class 2 | Class 3 |
| --- | --- | --- | --- | --- | --- | --- |
|  |  |  | 100 0  score score | Min Max | Min Max | Min Max |
| CRITERIA |  |  |  |  |  |  |

Topography % 0 10 0 1 1 4 4 10

Groundwater depth m 0 250 0 7 7 50 50 250

| Surface water distance | km | 0 | 5 | 0 | 0.5 | 0.5 | 2 | 2 | 5 |
| --- | --- | --- | --- | --- | --- | --- | --- | --- | --- |
| Market travel time | hr | 0 | 3 | 0 | 0.5 | 0.5 | 1 | 1 | 3 |
| Distance to existing LSI | km | 0 | 10 | 0 | 1 | 1 | 5 | 5 | 10 |
| SCORE |  |  |  |  |  |  |  |  |  |
| Topography | 1 |  |  | 100 | 90 | 90 | 60 | 60 | 0 |
| Groundwater depth | 2 |  |  | 100 | 100 | 100 | 68 | 68 | 20 |
| Surface water distance | 3 |  |  | 100 | 90 | 90 | 60 | 60 | 0 |
| Market travel time | 4 |  |  | 100 | 83 | 83 | 67 | 67 | 0 |
| Distance to existing irrigation | 5 |  |  | 100 | 80 | 80 | 60 | 60 | 0 |
| Max (2,3) |  |  |  | 100 | 100 | 100 | 68 | 68 | 20 |

# **Appendix D Descriptive Summary**

Table D1 Descriptive summary for Ethiopia (pooled sample)

Table D2 Descriptive summary for Tanzania (pooled sample)

**Reference:**

Mekonnen D., E. Bryan, J. Choufani, E. Davies, C. Ringler and S. Passarelli. 2019. A User Guide to the Innovation Lab for Small Scale Irrigation (ILSSI) Baseline Survey Data: Ethiopia & Tanzania. Washington, DC: International Food Policy Research Institute (IFPRI).
