# Supplementary material for: Irrigation improves weight‐for‐height z‐scores of children under five, and Women's and Household Dietary Diversity Scores in Ethiopia and Tanzania
Source: Matern Child Nutr. 2022 Jun 24;18(4):e13395. doi: 10.1111/mcn.13395 (PMC9480923; doi:10.1111/mcn.13395)
Supplement: Supplementary file 2 — Supporting information. [file MCN-18-e13395-s001.docx]

**Supplemental Table A1: Panel Fixed Effects Estimation of Access to Irrigation on HDDS and WDDS in Ethiopia**

|  | (1) | (2) |
| --- | --- | --- |
|  | Household dietary diversity score | Women's DD Score |
| Irrigation | 0.279 (0.222) | -0.171 (0.214) |
|  |  |  |
| Feb to April 2017  (compared to Dec 2014) | -0.079 (0.180) | -0.508^***^ (0.121) |
|  |  |  |
| Irrigation X Feb to April 2017 | -0.299 (0.228) | 0.488^*^ (0.210) |
|  |  |  |
| Drought occurred | 0.232 (0.176) | -0.395 (0.220) |
|  |  |  |
| Irrigation X Drought | 0.000 (0.258) | 0.258 (0.209) |
|  |  |  |
| Total Land size | 0.021 (0.171) | 0.079 (0.147) |
|  |  |  |
| Owns cows | 0.097 (0.190) | -0.018 (0.148) |
|  |  |  |
| Owns goats/sheep/pigs | 0.052 (0.115) | -0.081 (0.096) |
|  |  |  |
| Owns chickens/birds | -0.109 (0.150) | -0.032 (0.103) |
|  |  |  |
| Remittances | -0.381 (0.216) | -0.114 (0.236) |
|  |  |  |
| Self-employment income | 0.242^*^ (0.112) | -0.119 (0.127) |
|  |  |  |
| Storm/flood | 0.205 (0.201) | -0.143 (0.141) |
|  |  |  |
| Insect/disease damage | -0.126 (0.244) | -0.137 (0.174) |
|  |  |  |
| Illness/death in family | -0.087 (0.182) | -0.150 (0.156) |
|  |  |  |
| Log household size | 0.304 (0.309) | 0.933^**^ (0.289) |
|  |  |  |
| Constant | 5.14^***^ (0.619) | 2.19^***^ (0.479) |
| Observations | 950 | 950 |

Standard errors are in parentheses; ^*^ *p* < 0.05, ^**^ *p* < 0.01, ^***^ *p* < 0.001

**Supplemental Table A2: Panel Fixed Effects Estimation of Access to Irrigation on WDDS and HDDS in Tanzania**

|  | (1) | (2) |
| --- | --- | --- |
|  | Household dietary diversity score | Women's DD Score |
| Irrigation | 0.195 (0.328) | 0.358 (0.170) |
|  |  |  |
| July 2017  (compared to July 2015) | 0.828^**^ (0.245) | 0.0217 (0.223) |
|  |  |  |
| Irrigation X July2017 | -0.433^*^ (0.196) | -0.124 (0.178) |
|  |  |  |
| Drought occurred | -0.025 (0.279) | -0.424^*^ (0.171) |
|  |  |  |
| Irrigation X Drought | 1.09 (0.526) | 0.343 (0.350) |
|  |  |  |
| Total Land size | 0.118 (0.205) | 0.209 (0.110) |
|  |  |  |
| Owns cows | -0.479 (0.944) | 0.482 (1.069) |
|  |  |  |
| Owns goats/sheep/pigs | 0.281 (0.497) | 0.279 (0.443) |
|  |  |  |
| Owns chickens/birds | 0.191 (0.246) | 0.056 (0.279) |
|  |  |  |
| Remittances | 0.196 (0.301) | 0.101 (0.165) |
|  |  |  |
| Self-employment income | 0.036 (0.185) | 0.106 (0.189) |
|  |  |  |
| Gifts | 0.492 (0.325) | 0.246 (0.273) |
|  |  |  |
| Storm/flood | -0.042 (0.285) | -0.113 (0.170) |
|  |  |  |
| Illness/death in family | -0.329 (0.198) | -0.212 (0.169) |
|  |  |  |
| Log household size | 0.453 (0.220) | 0.404 (0.297) |
|  |  |  |
| Log woman's age | 0.304 (0.554) | -1.12 (0.778) |
|  |  |  |
| Woman's education | -0.037 (0.032) | 0.024 (0.030) |
|  |  |  |
| Constant | 3.42 (2.07) | 7.10^*^ (2.98) |
| Observations | 972 | 916 |

Standard errors are in parentheses; ^*^ *p* < 0.05, ^**^ *p* < 0.01, ^***^ *p* < 0.001

**Supplemental Table A3: Panel Fixed Effects Estimation of Access to Irrigation on WHZ, Wasting, HAZ, and Stunting in Ethiopia**

|  | (1) | (2) | (3) | (4) |
| --- | --- | --- | --- | --- |
|  | Weight-for-length/height Z-score | Wasting | Length/height-for-age Z-score | Stunting |
| Irrigation | 0.415 (0.534) | 0.108 (0.091) | 0.565 (0.623) | -0.083 (0.201) |
|  |  |  |  |  |
| Feb to April 2017  (compared to Dec 2014) | -0.579 (0.502) | -0.080 (0.075) | 2.04^***^ (0.414) | -0.259 (0.161) |
|  |  |  |  |  |
| Irrigation X Feb to April 2017 | 0.609 (0.530) | -0.051 (0.061) | -1.30^**^ (0.434) | 0.034 (0.144) |
|  |  |  |  |  |
| Drought occurred | -0.379 (0.718) | 0.336^*^ (0.131) | 0.185 (0.705) | -0.272 (0.254) |
|  |  |  |  |  |
| Irrigation X Drought | 0.627 (1.11) | -0.300^*^ (0.140) | -0.596 (0.790) | 0.377 (0.321) |
|  |  |  |  |  |
| 6 to 12 months old | -0.571 (0.501) | 0.059 (0.107) | 1.08^*^ (0.370) | -0.175 (0.162) |
|  |  |  |  |  |
| 12 to 24 months old | -0.505 (0.343) | 0.004 (0.076) | 0.999^***^ (0.150) | -0.053 (0.089) |
|  |  |  |  |  |
| Sick the previous 2 weeks | -1.20^*^ (0.439) | 0.084 (0.074) | -0.176 (0.318) | 0.150 (0.121) |
|  |  |  |  |  |
| Average birthweight | -0.531 (0.423) | -0.048 (0.073) | -0.116 (0.278) | -0.124 (0.135) |
|  |  |  |  |  |
| Below average birthweight | -0.453 (0.503) | -0.140 (0.074) | -0.911 (0.425) | -0.130 (0.151) |
|  |  |  |  |  |
| Siblings under 5 = 1 | -0.390 (0.297) | -0.074 (0.056) | -0.647^*^ (0.255) | 0.141 (0.133) |
|  |  |  |  |  |
| Siblings under 5 = 2 | 1.02 (1.17) | 0.280^*^ (0.130) | -1.93 (1.12) | 0.833 (0.549) |
|  |  |  |  |  |
| Exclusive breast feeding | -0.424 (0.249) | -0.046 (0.043) | -0.715^*^ (0.294) | 0.102 (0.096) |
|  |  |  |  |  |
| Total Land size | 0.114 (0.395) | 0.006 (0.062) | -0.068 (0.300) | 0.140 (0.116) |
|  |  |  |  |  |
| Owns cows | 1.52^**^ (0.393) | -0.118 (0.087) | 0.236 (0.368) | -0.098 (0.148) |
|  |  |  |  |  |
| Owns goats/sheep/pigs | 0.736 (0.444) | -0.008 (0.046) | 0.048 (0.191) | -0.073 (0.054) |
|  |  |  |  |  |
| Owns chickens/birds | -0.110 (0.227) | -0.043 (0.086) | 0.149 (0.179) | 0.188^*^ (0.069) |
|  |  |  |  |  |
| Remittances | -1.19 (0.952) | -0.018 (0.127) | 1.17^*^ (0.539) | -0.134 (0.241) |
|  |  |  |  |  |
| Self-employment income | -0.896 (0.451) | -0.090 (0.077) | 0.001 (0.387) | -0.102 (0.174) |
|  |  |  |  |  |
| Gifts | -0.124 (0.628) | -0.169 (0.102) | -0.197 (0.515) | -0.170 (0.255) |
|  |  |  |  |  |
| Illness/death in family | -0.048 (0.360) | 0.024 (0.111) | 0.800 (0.439) | -0.436^*^ (0.158) |
|  |  |  |  |  |
| Storm/flood | -2.54^***^ (0.492) | 0.035 (0.084) | -0.731 (0.539) | 0.496^**^ (0.134) |
|  |  |  |  |  |
| Insect/disease damage | -0.125 (0.515) | 0.093 (0.122) | -0.971 (0.506) | 0.101 (0.269) |
|  |  |  |  |  |
| Log household size | 0.429 (0.793) | -0.067 (0.194) | 0.106 (0.940) | 0.482 (0.283) |
|  |  |  |  |  |
| Woman's education | 0.010 (0.065) | -0.024 (0.015) | 0.008 (0.060) | -0.024 (0.019) |
|  |  |  |  |  |
| Constant | -0.978 (2.16) | 0.416 (0.388) | -2.61 (2.01) | -0.369 (0.695) |
| Observations | 468 | 468 | 461 | 461 |

Standard errors are in parentheses; ^*^ *p* < 0.05, ^**^ *p* < 0.01, ^***^ *p* < 0.001

**Supplemental Table A4: Panel Fixed Effects Estimation of Access to Irrigation on WHZ, HAZ, and Stunting in Tanzania**

|  | (1) | (2) | (3) |
| --- | --- | --- | --- |
|  | Weight-for-length/height Z-score | Length/height-for-age Z-score | Stunting |
| Irrigation | 0.368 (0.615) | -0.531 (0.375) | -0.081 (0.210) |
|  |  |  |  |
| Drought occurred | -0.034 (0.360) | 0.689 (0.437) | -0.101 (0.131) |
|  |  |  |  |
| Irrigation X Drought | 0.510 (0.544) | -0.198 (0.844) | 0.162 (0.279) |
|  |  |  |  |
| July 2017  (compared to July 2015) | 0.515 (0.548) | 0.809 (0.557) | -0.557^*^ (0.200) |
|  |  |  |  |
| Irrigation X July2017 | -0.450 (0.288) | 0.788 (0.597) | -0.266^**^ (0.084) |
|  |  |  |  |
| 6 to 12 months old | -0.488 (0.356) | 2.59^***^ (0.409) | -0.781^***^ (0.160) |
|  |  |  |  |
| 12 to 24 months old | -0.085 (0.207) | 1.53^**^ (0.474) | -0.479^*^ (0.174) |
|  |  |  |  |
| Sick the previous 2 weeks | -0.280 (0.201) | 0.313 (0.255) | 0.052 (0.130) |
|  |  |  |  |
| No. of siblings under 5 = 1 | 0.079 (0.113) | -0.257 (0.303) | 0.244^*^ (0.082) |
|  |  |  |  |
| No. of siblings under 5 = 2 | 1.31^**^ (0.337) | -2.16^*^ (0.952) | 0.824^**^ (0.237) |
|  |  |  |  |
| Exclusive breast feeding | -0.295 (0.163) | -0.050 (0.249) | -0.108 (0.087) |
|  |  |  |  |
| Total Land size | -0.242 (0.274) | 0.036 (0.326) | 0.243^*^ (0.099) |
|  |  |  |  |
| Owns goats/sheep/pigs | -0.462 (0.397) | 1.56 (0.778) | -0.235 (0.251) |
|  |  |  |  |
| Owns chickens/birds | 0.283^*^ (0.112) | -0.472 (0.334) | 0.137 (0.135) |
|  |  |  |  |
| Remittances | 0.349 (0.360) | -0.670 (0.701) | -0.056 (0.182) |
|  |  |  |  |
| Self-employment income | -0.043 (0.254) | 0.547 (0.270) | -0.193^*^ (0.081) |
|  |  |  |  |
| Gifts | 0.242 (0.293) | 0.386 (0.612) | -0.042 (0.295) |
|  |  |  |  |
| Illness/death in family member | -0.249 (0.118) | 0.185 (0.246) | -0.147 (0.070) |
|  |  |  |  |
| Storm/flood | 0.623^*^ (0.226) | -0.128 (0.331) | -0.213^*^ (0.090) |
|  |  |  |  |
| Log household size | -0.329 (0.355) | -0.152 (0.976) | -0.391 (0.375) |
|  |  |  |  |
| Woman's education | 0.050 (0.030) | 0.020 (0.042) | 0.010 (0.026) |
|  |  |  |  |
| Constant | 0.383 (0.667) | -2.20 (1.67) | 1.32 (0.712) |
| Observations | 459 | 453 | 453 |

Standard errors are in parentheses; ^*^ *p* < 0.05, ^**^ *p* < 0.01, ^***^ *p* < 0.001
